# Activated carbon N-acetylcysteine microcapsule protects against nonalcoholic fatty liver disease in young rats via activating telomerase and inhibiting apoptosis

**DOI:** 10.1371/journal.pone.0189856

**Published:** 2018-01-11

**Authors:** Tingting Shi, Xingxin Yang, Hongping Zhou, Jianjun Xi, Jingjing Sun, Yunling Ke, Jiankang Zhang, Yidan Shao, Xiaojie Jiang, Xuwang Pan, Shourong Liu, Rangxiao Zhuang

**Affiliations:** 1 Department of Pharmaceutical Preparation, The Hangzhou Xixi Hospital Affiliated to Zhejiang Chinese Medical University, Hangzhou, Zhejiang, China; 2 College of Pharmaceutical Science, Yunnan University of Traditional Chinese Medicine, Kunming, Yunnan Province, P.R. China; 3 Department of Pharmacy, Hangzhou Children’s Hospital, Hangzhou, Zhejiang, China; IDIBAPS Biomedical Research Institute, SPAIN

## Abstract

Non-alcoholic fatty liver disease (NAFLD) is becoming one of the world's most common chronic liver diseases in childhood, yet no therapy is available that has been approved by the food and drug administration (FDA). Previous studies have reported that telomere and telomerase are involved the development and progression of NAFLD. This study was designed to investigate the potential beneficial effects of activated carbon N-acetylcysteine (ACNAC) microcapsules on the development of NAFLD in young rats as well as the underlying mechanism(s) involved. Three-week old male Sprague Dawley rats were given high-fat diet (HFD) with/without ACNAC treatment for 7 consecutive weeks. Liver pathologies were determined by hematoxylin and eosin (H&E) and Oil Red O staining, as well as by changes in biochemical parameters of plasma alanine transaminase (ALT) and aspartate transaminase (AST) levels, respectively. Glucose homeostasis was evaluated by the glucose tolerance test and the liver telomere length and activity were measured by real time PCR and telomeric repeat amplification protocol (TRAP). Western blot analysis was performed to determine the expression level of Bcl-2, Bax and Caspase-3. Our results demonstrated that ACNAC supplementation improved liver pathologies of rats that received long-term HFD feeding. ACNAC supplementation prevented HFD-induced telomere shortening and improved telomerase activity. Moreover, in comparison to HFD-fed rats, ACNAC supplementation markedly increased the expression of Bcl-2, but significantly decreased the expression of Bax and Caspase-3 in juvenile rats. Together, these results indicate that ACNAC may be a promising choice for preventing and treating NAFLD among children.

## Introduction

In coastal areas of China, non-alcoholic fatty liver disease (NAFLD) has become one of the most common diseases among higher modern living standards, better dietary structure, accelerated aging and advanced medical and health undertakings[1,2]. Epidemiological data indicated that in foreign adults, the incidence of NAFLD is 20% to 33% [3], whereas in healthy children around the world the incidence is 3% to 10% [4]. The incidence of fatty liver in Chinese children is about 2% to 4% [5]. Most of NAFLD is caused by high-fat diet, and is common among smart phone addicts and individuals with limited exercise. It has been reported that roughly 8 million children in China suffer from NAFLD.

As in adults, NAFLD involves a series of liver pathological changes in children ranging from simple fatty liver degeneration to non-alcoholic steatohepatitis (NASH), liver fibrosis and liver cirrhosis [6–8]. In recent years, NAFLD has become one of the world's most common chronic liver diseases in childhood, which is evidenced by its incidence [9], and therefore has gained increased attention.

N-acetylcysteine (NAC) is a precursor of reduced glutathione, which can improve the biosynthesis of intracellular glutathione and inhibit the oxidation stress that is induced by the imbalance of oxygen free radicals and self-anti-oxidation[10,11]. In clinical applications, NAC exhibits good efficacy including anti-hepatic fibrosis, prevention and treatment of fatty liver, improvement of intrahepatic circulation, and abnormal liver function indexes [12–17], however NAC may leading to several adverse reactions such as high dosage, strong first-pass effects, low bioavailability and rapid metabolism. However, with the advantages of developed porous structures, high specific surface areas, good adsorption properties and biocompatibility [18], activated carbon for medical purposes have the potential to significantly facilitate the sustained release of compounds, thereby improving the action time and bioavailability [19]. In a previous study, ACNAC was prepared by effectively combining NAC with medicinal activated carbons through an orthogonal experiment [20–22]. In vitro and in vivo studies have showed that ACNAC may slow down the release of NAC, thereby improving the stability of NAC and enhance the anti-hepatic fibrosis and anti-oxidation effects in rats [12, 13, 23, 24]. Telomeres consist of stretches of repeated DNA sequences that are located at the ends of chromosomes and shorten during mitosis, and protect the tips of chromosomes[25]. Telomeres and telomerase play an important role in the onset and the progression of liver disease independent of the underlying etiology[26, 27]. The role of telomeres in the pathogenesis of liver disease may be explained as follow: in the case of NAFLD, trigger factors, such as obesity and insulin resistance, induce a condition of chronic hepatic damage and regeneration that is characterized by progressive hepatocytes telomere shortening and senescence [28]. Therefore, in the present study, we evaluated the effects of ACNAC on telomere and telomerase of high fat diet (HFD)-induced liver cells during the weaning period in Sprague Dawley (SD) rats with nonalcoholic steatohepatitis. We also investigated the mechanisms of ACNAC based on reduced dosage, less side effects and good efficacy regarding the physiologic features of children.

## Materials and methods

### Animals

Sixty four weaned male Sprague Dawley (SD) rats (45–60 g) were purchased from the animal center of Zhejiang Academy of Medical Sciences (animal license number SCXK (Zhe) 2014–0001). Throughout the experiments, rats were kept in an environment that was maintained at 22 ± 1°C with a 12 h light/12 h dark cycle and had access to a standard commercial diet and water ad libitum. All experiments were performed in accordance with the National Institutes of Health Guide for the Care and Use of Laboratory Animals, and approved by the Animal Care Committee of Xixi hospital affiliated to Zhejiang University of Traditional Chinese Medicine (Hangzhou, China).

After being administered fodder for 1 day, 3-week old rats were randomly divided into a normal (Normal) group, model (Model) group, polyene phosphatidylcholine (PPC) group, N-acetylcysteine (NAC) group, activated carbon microcapsule (AC) group, and an activated carbon N-acetylcysteine microcapsule low-dose, middle-dose and high-dose groups (ACNAC1, ACNAC2, and ACNAC3, respectively). The normal group was fed with a standard fodder, whereas the other groups were fed a HFD (69% of basic feed, 10% of lard oil, 2% of cholesterol, 5% of sugar, 0.5% of cholate, 10% of yolk powder, 3% of yeast powder and 0.5% of decavitamin). The low-dose, middle-dose and high-dose ACNAC groups were intragastrically administrated with NAC (20, 40 and 80 mg/kg). NAC and PPC group were intragastrically administrated 80 mg/kg NAC, whereas the model and normal control group were intragastrically administrated an equal volume of 0.9% normal saline. After grouping, rats were treated once a day for 7 consecutive weeks, and were sacrificed 24 h after the last administration. Rats were euthanized via exposure to a gradually increasing dose of isoflurane and carbon dioxide (CO_2_)[29]. Blood and liver tissues were immediately collected and stored at -80°C for future experiments. All efforts were made to minimize the number of animals and their suffering. According to previous/pilot experiments, the mean of ALT level in rats was (57.990 ± 8.431), therefore we estimated 4 rats were need to reach a statistical significance of *P* < 0.05 with an 90% propability.

### H&E staining

Sections were cut and dewaxed, stained with hematoxylin solution for 10 min at room temperature and incubated in 1% hydrochloric-alcohol solution. Then, the sections were cleaned using tap water, stained with eosin liquor for 8 minutes, and separated by 75%, 85%, 90%, 95%, 100% and 100% alcohol solutions into different colors for 2 minutes respectively. Next, the sections were placed into 2 bottles of OT biological clarifier for 10 minutes each and sealed with neutral gums for observation under microscope. Refer to the *Guidelines for the Diagnosis and Treatment of Nonalcoholic Fatty Liver Diseases* formulated by the Fatty Liver and Alcoholic Liver Disease Study Group of the Chinese Liver Disease Association for criteria of the degree of hepatic steatosis [30], where NAFLD activity score (NAS) is set as 0 to 8 points.

### ORO staining

Liver tissues were cured, dehydrated, embedded in optimal cutting temperature compound (OCT), sliced (8 to 10 μm thick), fixed (frozen sections were rewarmed for 10 min and rinsed three times with phosphate buffer saline (PBS) for 5 minutes each), stained (the sections were incubated in Oil Red O (ORO) liquid for 15 minutes, rinsed three times with PBS for 5 min each and stained in ORO liquid at 37°C for 2 h) and differentiated (sections were differentiated in 75% alcohol for 2 s, and then washed with water for 1 minute); then cell nuclei were stained (Harris hematoxylin for 2 min, washed with tap water, differentiated in 1% hydrochloric-alcohol solution for several seconds, washed with tap water, turned blue with ammonia and washed with water) and sealed. The pathological changes were observed under light microscope to evaluate the degree of steatosis.

### Biochemical analysis

Levels ALT, AST, TC, TG, HDL-C, LDL-C, FBG and FINS were determined according to the instructions of reagent kits (Nanjing Jiancheng Bioengineering Institute, Nanjing, China). The insulin resistance index was calculated according to the formula FBG × FINS / 22.5[31].

### Insulin sensitivity test

The insulin sensitivity was tested by the steps of separating femoral vein from anesthetized rats, inserting detaining needles, injecting glucose and insulin with a micro-injector (at constant rate), monitoring the basal blood glucose and fasting blood glucose and calculating glucose infusion rate (GIR) within 60 to 120 min. The smaller GIR represents server insulin resistance, indicating the insulin sensitivity of rats.

### Liver index

The liver index was obtained by the steps of weighing anesthetized rats in grams, extracting fresh liver tissue and drying and weighing the wet weight of fresh liver tissue in grams and calculating according to the formula liver wet weight/body weight x 100%.

### Liver tissues extracted and the telomere length measurement by qPCR

① DNA was extracted from liver tissues and the OD_260_ to OD_280_ ratio obtained was between 1.7 and 1.9;

②The primer sequences are listed in Table 1

**Table 1:** List of primers for QPCR analysis.

The Primer	Sequence 5’-3’
tel-F	*CGGTTTGTTTGGGTTTGGGTTTGGGTTTGGGTTTGGGTT*
tel-R	*GGCTTGCCTTACCCTTACCCTTACCCTTACCCTTACCCT*
RatDNA-Actin-F	*GAGACCTTCAATGTGCCAGCAATGTA*
RatDNA-Actin-R	*GCGAGACACCATCACCACTATCCAAA*

③ Reaction conditions and system:

Reaction conditions include 94°C for 1 minute, 95°C for 10 seconds, 59°C for 10 seconds and 72°C seconds (40 cycles).

Reaction system (20 μl) includes 10 μl 2 × SYBR premix, 1 μl upstream primer, 1 μl downstream primer, 1 μl template and 7 μl ddH_2_O.

④ Test results

### Activity of telomerase in liver tissue

① Lysis: the well-grounded tissue samples were added to 200 μl NP-40 buffer lysis solution, placed on ice for 30 minutes and centrifuged at 12,000 rpm under 4°C for 20 minutes. The supernatant was extracted for use.

②Amplification

Primer sequences:

the reverse primer (ACX): *GCGCGGCTTACCCTTACCCTTACCCTAACC*

the internal standard (NT): *ATCGCTTCTCGGCCTTTT*

the substrate for 36-bp internal standard control (TSNT): *AATCCGTCGAGCAGAGTTAAAAGGCCGAGAAGCGAT*

TS: *AATCCGTCGAGCAGAGTT*

③Amplification conditions: 25°C for 40 min for extension. 95°C for 5 min to deactivate telomerase. 30 cycles at: 95°C for 30 sec, 52°C for 30 sec, 72°C for 45 sec. 72°C for 10 min.

④Running acrylamide gel: Gel preparation, Electrophoresis detection, Ethidium bromide dyeing.

⑤Results analysis.

### Western blot analysis

① Total protein extraction: total protein was extracted by the steps of placing 200 mg liver of juvenile rats in 1.5 ml eppendorf (EP), adding 1ml radio immunoprecipitation assay (RIPA) lysis solution on the ice and homogenizing until full lysis, centrifuging at 12,000 rpm under 4°C for 5 min and extracting the supernatant. Then the supernatant was stored in refrigerator at -80°C. The bicinchoninic acid (BCA) method was used to test the protein concentration of the sample; ②sodium dodecyl sulfate polyacrylamide gel electrophoresis (SDS-PAGE) electrophoresis was performed by the steps of a, preparation of polyacrylamide gel, 5% stacking gel and 12% separating gel; b, sample application, namely adjusting sampling amount of each gel point to 20 μg; c, electrophoresis, namely 60 mA cross-flow electrophoresis; ③ film conversion, namely 100V constant voltage electrophoresis for 65 min; ④ overnight stay in the blocked state of 5% skim milk powder; ⑤ primary antibody reaction, namely placing polyvinylidene fluoride (PVDF) film into blocking liquid at proper concentration (with dilution ratio of 1:100), and shaking in a shaker at the room temperature for 2 h; ⑥ washing, namely discarding the blocking liquid and antibody, rinsing the filter with phosphate buffered saline with tween-20 (PBST) film 3 times for 10 min each time and washing with tris buffered saline (TBS) for 10 min each time; ⑦ secondary antibody reaction, namely placing PVDF film into blocking liquid at proper concentration, and shaking in shaker at the room temperature for 1 h; ⑧ washing, namely washing with tris buffered saline with tween-20 (TBST) solution 3 times for 10 min each, and then washing with TBS solution once for 10 min; and ⑨coloration, namely preparation of diaminobenzidine (DAB) coloration liquid when needed. The PVDF film was washed and placed into the color reaction solution for 3 min (for close observation), the reaction was terminated with water if needed. The film was imaged with a full‑automatic digital gel image analysis system (Shanghai Tanon Technology Co., Ltd., Shanghai, China) and the results were analyzed with Image J version 1.48 (National Institutes of Health, Bethesda, USA).

### Statistical analysis

Measurement data are presented as the mean ± standard deviation (SD). Statistical analysis weres performed with SPSS software version 21 (SPSS, Inc., Chicago, IL, USA). One-way ANOVA was used for comparison of multiple groups, and the least sighnificant difference (LSD) method was used for pairwise comparisons. *P*<0.05 was considered statistically significant.

## Results

### 1. ACNAC administration attenuates liver pathologies of NAFLD in rats

**Fig 1:**
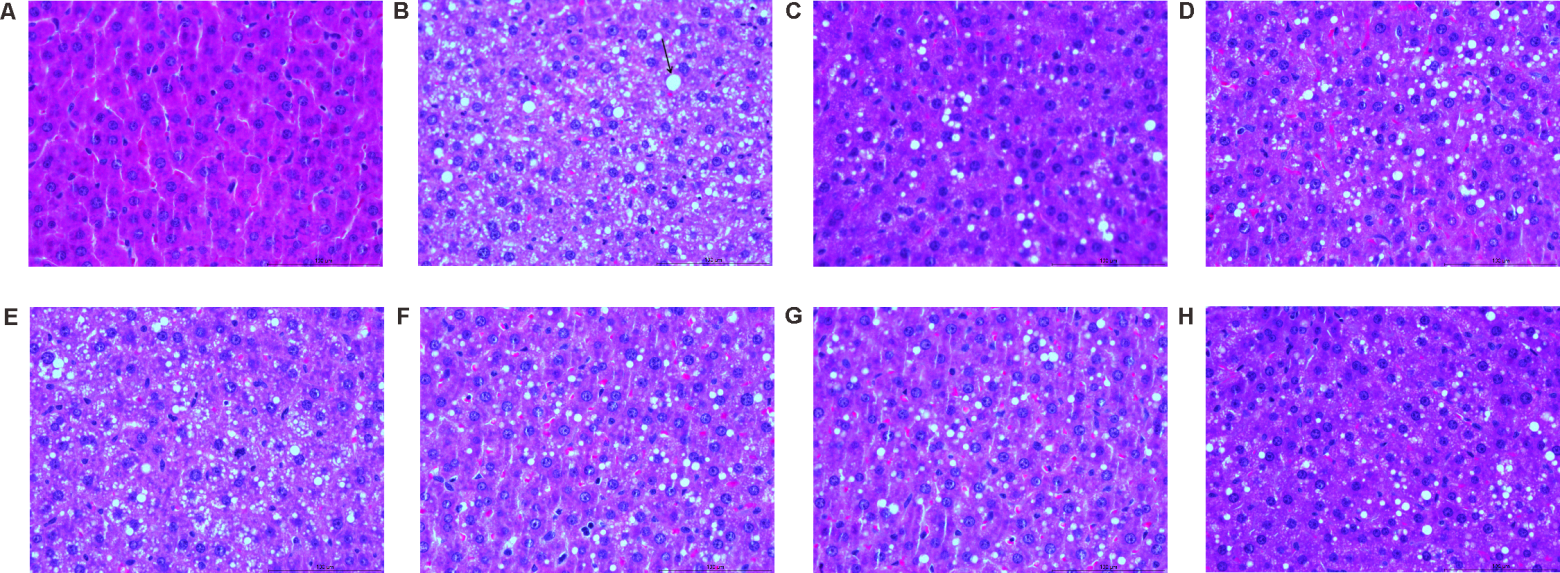
Pathological changes in rat liver tissue indicated by H&E staining. Nonalcoholic steatohepatitis (NASH) (hepatic steatosis and inflammation with hepatocellular damage [ballooning] with/without fibrosis) [57]. (A) Normal group, (B) Model group, (C) Polyene phosphatidyl choline group, (D) N-acetylcysteine control group, (E) Activated carbon release microcapsule control group, (F) N-acetylcysteine activated carbon release microcapsule low dose group, (G) N-acetylcysteine activated carbon release microcapsule middle dose group, (H) N-acetylcysteine activated carbon release microcapsule high dose group.

**Fig 2:**
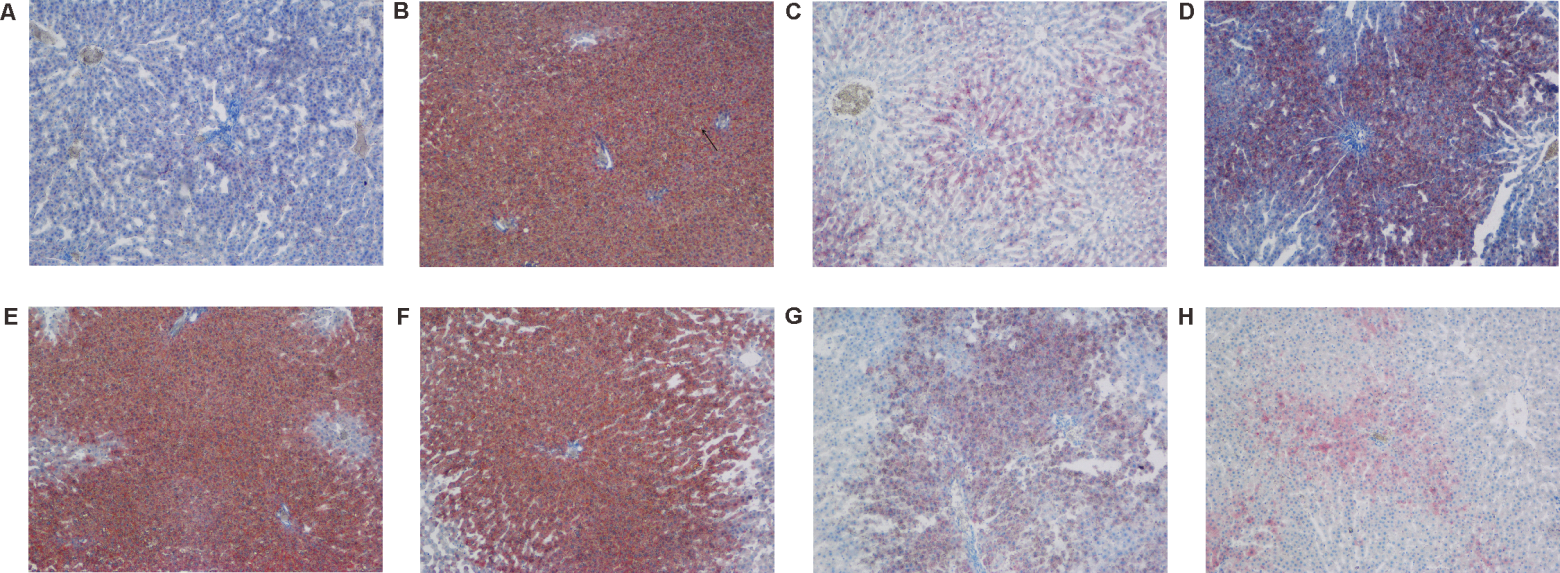
Lipid droplets in liver cells of SD rats after ORO staining. (A) normal group, (B) model group, (C) polyene phosphatidyl choline group, (D) N-acetylcysteine control group, (E) activated carbon release microcapsule control group, (F) N-acetylcysteine activated carbon release microcapsule low dose group, (G) N-acetylcysteine activated carbon release microcapsule middle dose group, (H) N-acetylcysteine activated carbon release microcapsule high dose group.

Professional pathologists classified the hepatic steatosis and inflammation in the hepatic lobules according to the results of H&E and ORO staining in liver tissues. H&E staining results: The normal group showed integral hepatic lobule structure, radial arrangement of liver cells around the center of central vein, clear boundaries of liver cells, nucleus in the central cell and clear outline of hepatic lobules (Fig 1A), while in the model group, the liver cell volume was significantly increased, mild and moderate to severe steatosis and ballooning were observed in liver tissues, vacuoles of different sizes were filled in the cells, and some inflammatory cells and focal necrosis were observed in some areas(Fig 1B). The AC group and the model group behaved similarly(Fig 1E). In comparison to the model group, hepatocellular steatosis and inflammation degree were reduced to varying degrees (Fig 1G and 1H) in the ACNAC groups; while ACNAC1 groups was less obvious (Fig 1F). The PPC group and the ACNAC3 group behaved similarly(Fig 1C). ORO staining results: The normal group showed pale blue liver and no significant color of lipid droplets(Fig 2A); while the model groups showed red liver cells and dense distribution of lipid droplets in most rats(Fig 2B). The AC group and the model group behaved similarly(Fig 2E). In addition, red dye was stronger in the high-dose arm of liver cells in the ACNAC groups (Fig 2H); while the low-dose and middle-dose groups were weaker(Fig 2F and 2G). The PPC group and the ACNAC3 group behaved similarly(Fig 2C). It was founded that the model group reached the NASH grade while ACNAC groups showed different degrees of decline, suggesting that ACNAC treatments was beneficial for NAFLD development.

### 2. Long-term HFD consumption of rat is a well-established animal model of NAFLD

**Fig 3:**
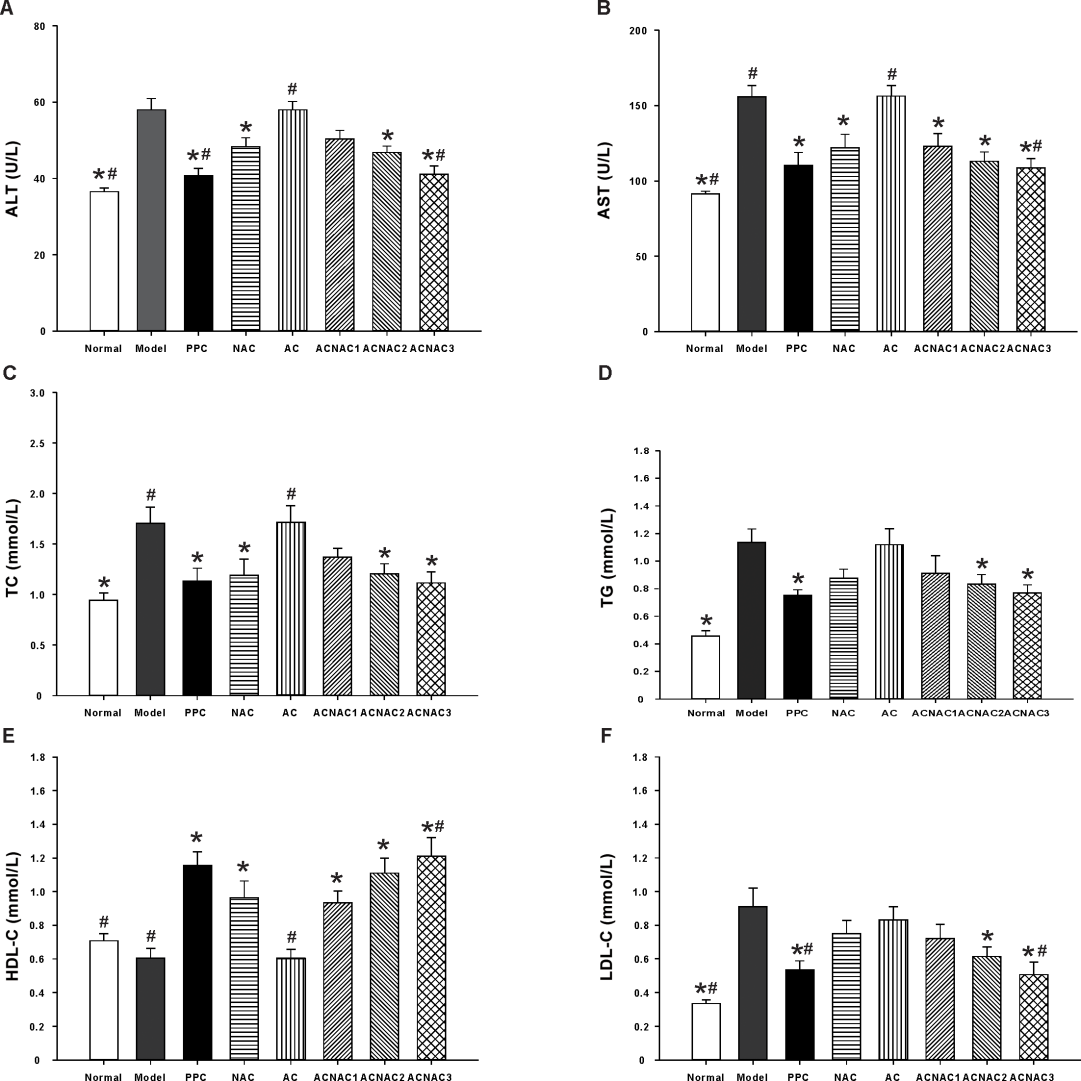
ALT, AST, TC, TG, LDL-C, HDL-C in SD rats serums. (A) ALT, (B) AST, (C) TC, (D) TG, (E) HDL-C, (F) LDL-C. ALT, AST, TC, TG, HDL-C and LDL-C were determined as described in materials and methods. Bars represent the means ± SD of results obtained with per experimental group. *P < 0.05, vs. the Model group, ^#^*P* < 0.05, vs. the NAC group, as determined by one way analysis of variance and LSD method. ALT, alanine transaminase; AST, aspartate transaminase; FBG, fasting blood glucose; FINS, insulin resistance.

As expected, a 7-week high fat diet (HFD) feeding induced early-stage liver pathologies of NAFLD in rat, including fatty liver and liver injury, which is manifested by increased plasma ALT, AST, TC, TG, LDL-C levels (Fig 3F) and decreased plasma HDL-C levels (Fig 3E) respectively. Compared with the model group, ACNAC, especially in the high-dose arm, PPC, significantly decreased activity of AST and ALT as well as contents of TC, TG and LDL-C, whereas promptly elevated content of HDL-C (Fig 3). Compared with the NAC group, ACNAC3 significantly decreased activity of AST and ALT as well as content of LDL-C, whereas promptly elevated content of HDL-C (Fig 3). Furthermore, no significant differences were observed in the AST, ALT, TC, TG, and LDL-C levels between the ACNAC high-dose and PPC groups (Fig 3). Taken together, these results indicate that ACNAC improves liver pathologies (H&E and Oil red O staining), ALT, AST, and other plasma indexes.

### 3. ACNAC administration improves whole-body glucose homeostasis in HFD-fed rats

**Fig 4:**
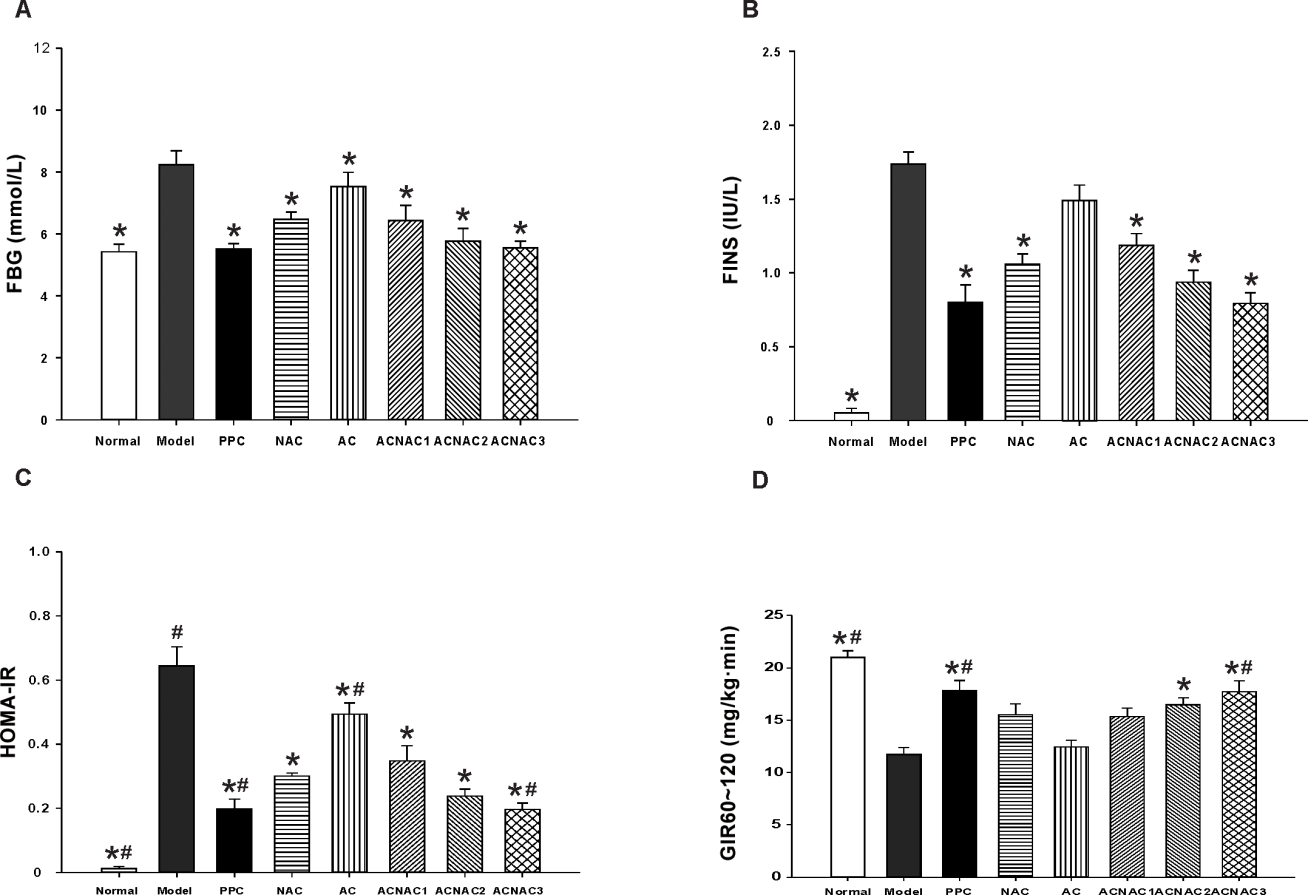
FBG, FINS, HOMA-IR and GIR_60~120_ in SD rats serums. (A) FBG, (B) FINS, (C) HOMA-IR, (D) GIR_60~120_. FBG, FINS, HOMA-IR and GIR_60~120_ were determined as described in materials and methods. Bars represent the means ± SD of results obtained with per experimental group. *P < 0.05, vs. Model group, ^#^*P* < 0.05, vs. the NAC group, as determined by one way analysis of variance and LSD method. FBG, fasting blood glucose; FINS, fasting insulin; HOMA-IR, insulin resistance index; GIR_60~120,_ glucose infusion rate (GIR) in 60 to 120 min.

**Fig 5:**
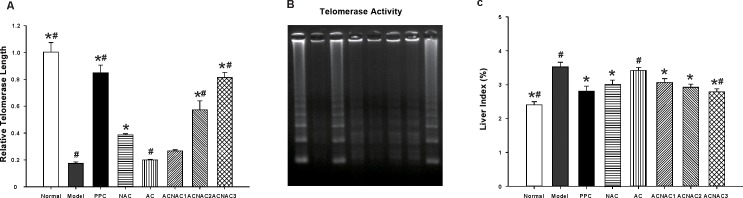
Quantitative RT-PCR and TRAP of telomere length and the telomerase activity in liver tissues of SD rats. A, Telomere length; B, Telomerase activity; C, Liver index. Normal, normal group; Model, model group; PPC, polyene phosphatidyl choline group; NAC, N-acetylcysteine control group; AC, activated carbon release microcapsule control group; ACNAC1, N-acetylcysteine activated carbon release microcapsule low dose group; ACNAC2. N-acetylcysteine activated carbon release microcapsule middle dose group; ACNAC3, N-acetylcysteine activated carbon release microcapsule high dose group. Bars represent the means ± SD of results obtained with per experimental group. *P < 0.05, vs. the Model group, ^#^*P* < 0.05, vs. the NAC group, as determined by one way analysis of variance and LSD method.

NAFLD is considered as the hepatic manifestation of the metabolic syndrome (MS), since hepatic fat accumulation is a consequence of systemic insulin resistance and hyperinsulinemia. Insulin plays an essential role in the progression of NAFLD, however, the exact mechanisms underlying insulin resistance in NAFLD remain to be clearly defined. The observations from animal studies above spurred us to examine the fasting blood glucose (FBG), fasting insulin (FINS), insulin resistance index (HOMA-IR) and liver index (LI) in NAFLD development. Interestingly, we observed that compared to the NAC group, ACNAC3 significantly decreased HOMA-IR, and LI, but increased GIR_60~120_ (Figs 4 and 5C) Further analysis indicated that ACNAC treatment induced a serious of biological effects such as a lower blood sugar, regulates lipid metabolism, improves insulin resistance, and reduces liver index. Therefore, it is worthwhile to further study the role of ACNAC in NAFLD. Furthermore, no significant differences were observed in the FBG, FINS, HOMA-IR, and LI levels between the ACNAC high-dose and PPC groups (Figs 4 and 5C).

### 4. ACNAC administration prevents HFD-induced hepatic telomerase suppression and telomere length shortening

In the context of liver injury, shortened telomere length leads to an impaired regenerative capacity of hepatocytes and an increased cirrhosis development. Agreement with that, telomerase activity was associated with NAFLD progression. Given crucial importance of telomeres and telomerase in liver injury, a better understanding of telomere and telomerase biology might translate into new therapeutic strategies for patients with NAFLD. Therefore, future studies should evaluate the usefulness of telomere and telomerase as prognostic markers for NAFLD development. To further confirm this observation, QPCR was used to measure telomere length and TRAP to test telomerase activity of liver cells in NAFLD development. Interestingly, we observed that compared with the normal group, mRNA levels of telomere notably declined and the telomerase activity significantly decreased in the model group; further studies showed that, compared with the NAC group, ACNAC3 significantly increased the mRNA levels of telomere and notably promoted the telomerase activity (Fig 5A and 5B). Together, these results indicate that ACNAC shortened telomere length and reduced telomerase activity of liver cells in NAFLD development. Furthermore, no significant differences were observed in the telomere length, telomerase activity between the ACNAC high-dose and PPC groups (Fig 5A and 5B).

### 5. ACNAC administration prevents HFD-triggered anti-apoptotic Bcl-2 expression and pro-apoptosis Bax expression, leading to reduced Bcl-2/Bax ratio in the liver

**Fig 6:**
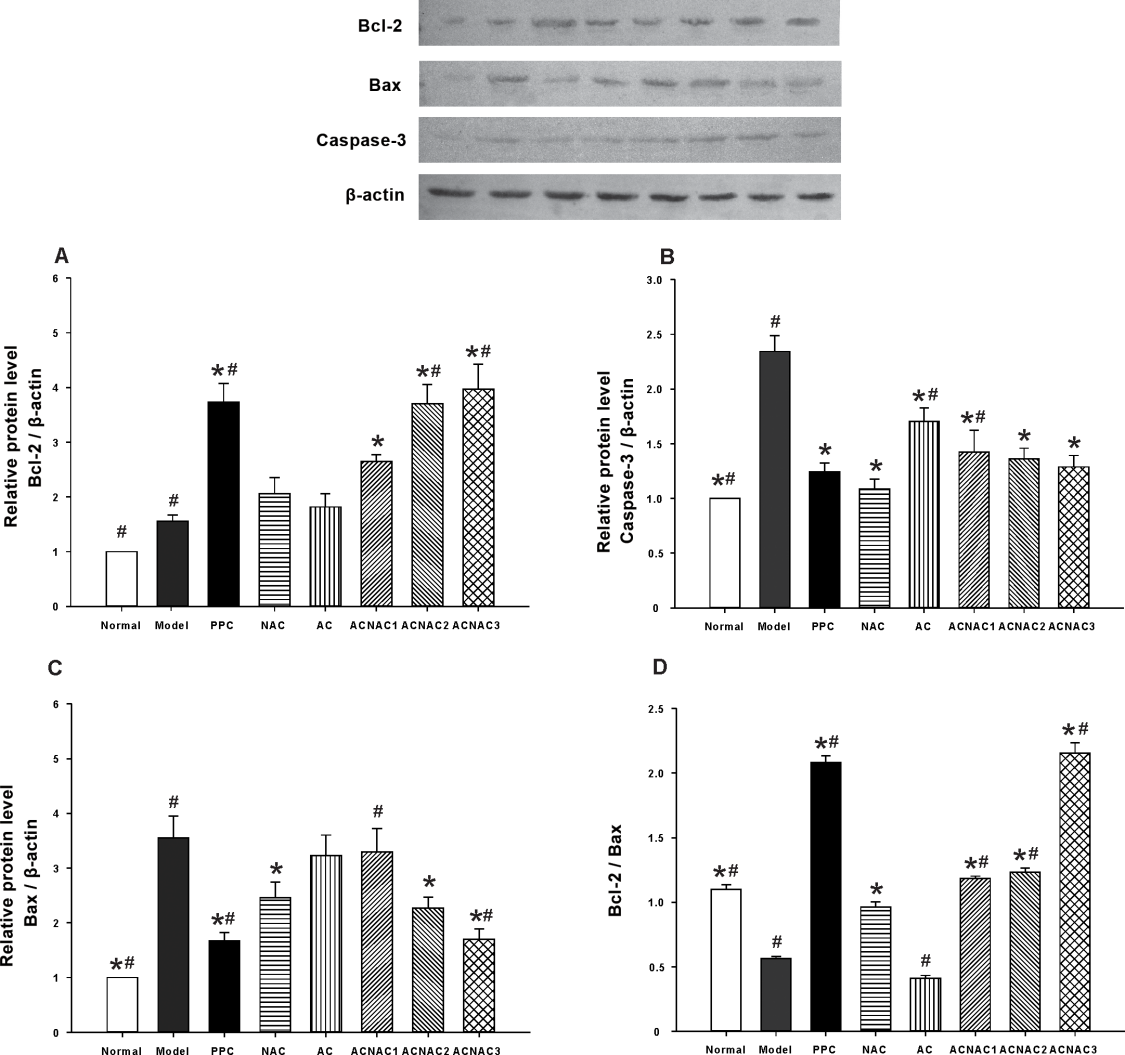
Bcl-2, Bax and Caspase-3 expression. The liver tissue relative protein content of (A) Bcl-2, (B) Caspase-3 and (C) Bax. Compared with the model group, the expression of Bax and Caspase-3 were decline in the ACNAC groups, but Bcl-2 was increased, Bars represent the means ± SD of results obtained with per experimental group. *P < 0.05, vs. the Model group, ^#^*P* < 0.05, vs. the NAC group, as determined by one way analysis of variance and LSD method.

With improvements of living standards and changes in lifestyle, the incidences of obesity and diabetes increased, thus the NAFLD incidence also increased in China. Although the pathogenesis of NAFLD is still unclear, there is evidence that liver cell apoptosis and related factors, such as Fas/FasL and Bcl-2/Bax, play important roles in NAFLD genesis and development. In this study, we established an improved high fat diet during the weaning period of NAFLD rat model to observe the expression of Bcl-2, Bax, Caspase-3 in liver tissue, in order to further clarify their roles and relationship in liver disease pathogenesis. Comparison to control diet, HFD consumption resulted in significantly increases in Bcl-2, Bax and Caspase-3, but reduced Bcl-2/Bax. At the same time, we found the study that compared to the NAC group, Bax was significantly decreased in the ACNAC3 group, but Bcl-2 and Bcl-2/Bax increased. Our result clearly demonstrated that ACNAC feeding suppressed the effect of HFD consumption on the expression of Bcl-2, Bax and Caspase-3, as well as Bcl-2/Bax (Fig 6). Furthermore, no significant differences were observed in the Bcl-2, Bax and Caspase-3 between the ACNAC high-dose and PPC groups (Fig 6).

## Discussion

Child-specific medicines are generally insufficient in China and in many other places in the world. There are many safety hazards as children are being told to take a smaller dosage of adult medicines. Combined with the difference between parents in urban and rural areas as well as lack of relevant clinical studies on the applicability of adult medicines in children, physicians must be rather careful when prescribing for children. Amongst the limited suitable varieties, dosage forms and specifications for children in China deserved more attention.

Previous studies on NASH in children prioritized on mechanisms related to lipid metabolism, insulin resistance, oxidative stress, however knowledge about telomeres is lacking. In this study, we investigated the effects of ACNAC on telomere length and telomerase activity in juvenile rats with NASH and the mechanisms for expressions of Bcl-2, Bax and Caspase-3.

Telomeres and telomerase play an important role in the onset and progression of liver disease and are independent of the underlying etiology. However, the role of telomere attrition and cell senescence is likely magnified in NAFLD where genetic risk factors and ageing have a major impact on the predisposition to advanced liver damage in combination with acquired risk factors [32]. Telomeres are repeated DNA sequenceS at the end of a linear chromosome, and form a a special structure at the end of the chromosome of eukaryotes. Telomeres can prevent the fusion between normal chromosome ends and ensure the integrity of each chromosome. In normal human cells, telomeres can shortened when a cell divides. Since the direction of DNA replication is in the 5' to 3' direction, the telomere shortens each time when DNA replicates. *In-vitro* studies have shown that 50 to 200 nucleotides are lost each time a cell divide, and that approximately 4,000 nucleotides are lost until the death of each cell [33, 34]. Therefore, telomere length, known as the “mitotic clock” for the lifespan of cells, reflects the cell replication history and replication potential [35]. Thus, telomeres are not only related to the stability of chromosomes, but are also involves in cellular senescence and death as well as in the occurrence and development of fatty liver, tumor, diabetes, metabolic syndrome and other diseases [28,36].

Telomerase, an enzyme responsible for telomere elongation in cells, is a typical nucleoprotein reverse transcriptase as telomere DNA sequence can be added to the end of eukaryotic chromosome[37]. Telomeres play an important role in maintaining chromosome stability and cell activity in different species. Telomerases may elongate telomeres, thereby improving the proliferation capacity of cells *in vitro*. Moreover, telomerase may well keep the genome integrity, telomere stability, long-term activity of cells. Telomerase functions by remedying the defects of DNA replication, which may prevent telomere loss from cell division and increase the number of cell divisions.

Previous studies have shown that [38] telomerase, which depends on physiological functions of telomere, may facilitate activation of stem cells, anti-apoptosis and anti-oxidative stress, thereby enhancing cell viability. Moreover, telomeres are shortened in liver cells in the process of liver fibrosis, so that the liver cells can be regenerated if telomerase is more active. Thus, the formation of liver fibrosis may be reduced or locked by improving the activity of telomerase in liver cells.

The main ingredients of PPC capsules are PPC, B vitamins, E vitamins. Polyenoic phospholipid is necessary for physiological processes of the body [39]. It can combine with liver cell and its organelle and become part of cell biological membrane. Moreover, it can repair the biological structure of a damaged liver cell membrance to maintain mobility and stability of liver cell membrane and recover damaged liver cells and transaminase. Also, it can reduce oxidative stress and lipid peroxidation, inhibit hepatocyte apoptosis, reduce hepatic stellate cell activation after inflammatory reaction, significantly lower transaminase level, effectively prevent liver cell degeneration and inflammatory fibrosis [40–42], thereby protecting liver cells. In addition, it can affect lipid metabolism process in the body, promote the formation of numerous cell soluble plasmids and rapid decomposition of fat in the body, ultimately inhibiting the accumulation of fat in the body. Therefore, PPC plays a role in numerous aspects, including anti-inflammation, antioxidation, and immune regulation function [43,44]. In clinic, PPC is widely used as a treatment in various types of liver disease [45]. The treatment of PPC has a good treatment effect on NAFLD and no adverse reactions are observed[46,47]. Therefore, PPC was used as a positive control drug.

Our results indicated that compared with the model group, the ACNAC groups, especially the high-dose arm, decrease the activity of ALT and AST and levels of TC, TG, LDL-C, FBG and FINS as well as HOMA-IR and liver index. Moreover, levels of HDL-C and GIR_60~120_ were increased. H&E and ORO staining showed that, the ACNAC groups had significantly lower livers of hepatic steatosis and inflammation compared with the model group. The ACNAC groups, especially the high-dose arm, elongate the telomere length and increase the telomerase activity. We found that compared with the NAC, the ACNAC3 had better curative effect in the treatment of NAFLD. Studies have revealed that hepatocyte apoptosis plays a key role in the progress of NAFLD to nonalcoholic steatohepatitis (NASH), liver fibrosis, cirrhosis and liver cancer [48, 49]. The occurrence of hepatocyte apoptosis is mainly regulated by death receptor signal transduction pathways, mitochondria signal pathways, and the endoplasmic reticulum signaling pathway [50, 51].

Although the pathogenesis of NAFLD is still not very clear, there is evidence that liver cell apoptosis-related factors such as Bcl-2/Bax, and Caspase all play important roles in NAFLD genesis and development [52, 53].

To investigate whether apoptosis-related factors are involved in the changes of telomere length and telomerase activity, we explored Bcl-2 gene and Caspase family. Western blot analyis indicated that, the ACNAC groups had relatively lower expression of Bax and Caspase-3 protein and higher expression of Bcl-2 compared with the model group. These findings were consistent with previously reported results. Bcl-2 is a key gene that determines hepatocyte apoptosis as it may play an important role in anti-apoptosis by activating a series of downstream genes in liver cells. When Bcl-2 expresses increased, the hepatocyte apoptosis may be inhibited. Bax gene is a key pivot in the process of apoptosis [54,55]. The ratio of Bcl-2 to Bax is called the “apoptosis switch” as cell apoptosis occurs when the Bax protein is dominant. Caspase family is an important protease in the process of apoptosis, which is finally achieved through the activation of Caspase. Moreover, the Caspase family is activated as cascade reaction, where Caspase-3 is the most critical protease responsible for mediating and executing death instructions. Li et al. [56] demonstrated that the intervention of Caspase-3 activation inhibited the occurrence of apoptosis. ACNAC inhibited hepatocyte apoptosis by upregulating the expression of Bcl-2 and downregulating the expression of Bax and Caspase-3. Thus, N-acetylcysteine packed into microcapsule protects aginst NAFLD in rats and had a positive meaning for the treatment of NAFLD.

This process may be associated with the control mechanism of telomere length and telomerase activity, which needs to be further explored. Hence, it can be primarily concluded that ACNAC can prevent SD rats induced from the weaning period from suffering NAFLD, which further provides a theoretical basis for research of NAFLD in children.

Thus, the above results indicated that in liver tissue, HFD was involved in the dysfunction of NAFLD and an improvement function may contribute to the hepato protective effects of ACNAC in NAFLD. Thus, ACNAC-focused research has a high significance. We believe that this study will provide the theoretical basis for clinical medication and will contribute to reduce the incidence of NAFLD in China.

## Abbreviations

NAFLD: Non-alcoholic fatty liver disease
ACNAC: activated carbon N-acetylcysteine
ALT: alanine transaminase
AST: aspartate transaminase
FBG: fasting blood glucose
FINS: insulin resistance
HOMA-IR: insulin resistance index
GIR_60~120_: glucose infusion rate in 60 to 120 min
TC: serum total cholesterol
TG: triglyceride
HDL-C: high-density lipoprotein
LDL-C: Low-density lipoprotein
Bcl-2: B-cell lymphoma-2
Caspase-3: cysteinyl aspartate specific proteinase 3
